# The *Neon Fruit Illusion*: A Fresh Recipe for Colour Science Demonstrations

**DOI:** 10.1177/0301006618824484

**Published:** 2019-02-07

**Authors:** Joshua Harvey, Takuma Morimoto, Manuel Spitschan

**Affiliations:** Department of Experimental Psychology, University of Oxford, UK; Department of Engineering Science, University of Oxford, UK; Department of Experimental Psychology, University of Oxford, UK

**Keywords:** colour, visual illusions, public engagement

## Abstract

At this year’s European Conference on Visual Perception, we debuted a novel colour science demonstration—and visual illusion—for the *Un mare di illusioni* exhibition. Under carefully curated lighting conditions, cycling through different illuminant spectra, certain fruits and vegetables appear to glow and dim in an unchanging environment. Encouraged by the positive reactions it received, and the numerous and specific questions from conference delegates, we here describe what this illusion is, why we believe it may work, and how this particular low-cost setup may be assembled and demonstrated for the amazement of your friends, students, and members of the public.

Human colour vision is remarkably stable across a range of conditions. One of the mechanisms underpinning this stability is colour constancy, when objects do not change their colour appearance despite changes in environmental lighting, even when the objects’ reflected spectra may change considerably. This is a significant achievement of the human visual system, as colour constancy is a mathematically ill-posed and under-constrained problem. Our novel *neon fruit illusion* capitalises on this, exploiting the physical conditions of lighting changes when colour constancy inevitably breaks down.

The illusion relies on both the dimensionality reduction from spectral to trichromatic information and the distinct reflectance properties of different objects. Two illuminant spectra may themselves be metameric, yielding the same amount of cone excitations and therefore causing no noticeable change in lighting. Depending on their respective reflectance functions, objects in a given scene may also yield the same amount of cone excitations under the two illuminants, therefore not exhibiting a change in luminance or chromaticity. The *neon fruit illusion* occurs when a largely nonnoticeable modulation in illumination causes a pronounced change in the appearance of *some* objects in the scene, while others remain constant. A reasonable inference from this stimulus is therefore that the object itself is emitting light or glowing.

In fact, the illuminants need not be nominally metameric, as long as the perceptual difference between them is very small relative to the change in *glowing* objects. An effective and affordable setup illustrates this effect using a commercially available four-primary LED bulb. If the scene is flooded with its relatively narrowband green channel, adding small amounts of the red channel will not noticeably affect the chromaticity of the light or the colour appearance of most objects (if they are white, yellow, green, blue, etc. under white illumination). However, reddish objects that were previously *in the dark* will exhibit a strong change in luminance, transitioning from dark grey/black to brightening red. Spectral measurements from this setup are shown in Figure 1. Observers will typically ascribe this colour change not to the lighting but to the objects themselves, either due to glow or colour change. This is not an impossibility—The objects could indeed have light sources buried within them; it therefore meets the criteria of an illusion suggested by Todorović (2018).

**Figure 1.**
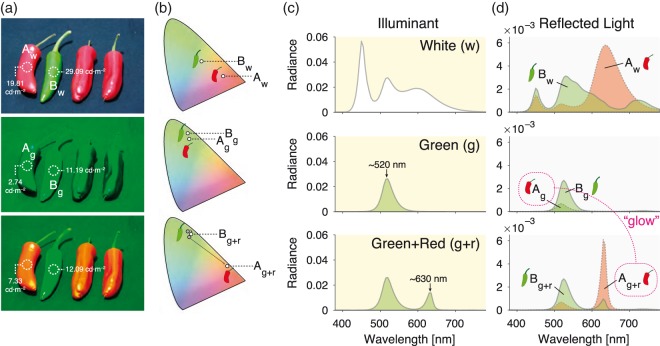
(a) Typical stimuli, in this case an assortment of red and green peppers, under white, green, and green + red LED illumination. The change between the latter two illuminants causes a pronounced increase in the luminance of the red peppers, while the green peppers show little change. (b) The chromaticity of the red peppers also shows a substantial shift. The spectra of different illuminant conditions (c) and the measured reflected spectra of the peppers (d) shown in panel (a) are also shown. Spectra are given in the online supplement. Radiance given in mW/cm^2^/sr/nm; measurements performed with a PR-670 spectroradiometer (Photo Research, Chatsworth, CA).

These manipulations do not merely cause changes in the apparent surface colour but can evoke a strong sensation of glow (where an object appears to emit light; Katz, 1935/2013). This phenomenon has been investigated in past studies with relatively simple stimuli (Bonato & Cataliotti, 2000; Bonato & Gilchrist, 1999; Speigle & Brainard, 1996; Uchikawa, Koida, Meguro, Yamauchi, & Kuriki, 2001; Yamauchi & Uchikawa, 2000, 2005). It is worth noting that such a sensation can occur even for natural objects, despite our priors that peppers should not be emitting light.

Interestingly, when shown as still images such as in Figure 1, the impression of glow weakens or may be entirely absent. It may therefore be the dynamic manipulation of spectral illumination that evokes strong sensations of glow in this case, and temporal modulation may be a fruitful avenue of research for further investigations into perceived glow.

We debuted such a setup at the *41st European Conference on Visual Perception* evening exhibition *Un mare di illusioni*. Sourcing a range of fresh fruits and vegetable from the local DESPAR supermarket in Trieste, we obtained fruits and vegetables with a broad range of natural reflectances. We set up shop in our own private room within the dock warehouse Magazzino 42, which gave us excellent control of the lighting. The fruits and vegetables were placed on a piece of standard blackout cloth on a table and illuminated by four LiFX bulbs (LiFX A60 LED Light) under software control (IFTTT running on an Android smartphone). The resultant exhibit is shown in frames of a video recorded on a mobile phone (without any post-processing) in Figure 2, and displayed in Figure 3 as a video-slice diagram.

**Figure 2.**
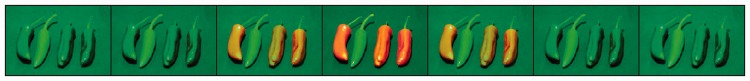
A sequence of frames taken from a video of the neon fruit illusion. Video available in the online supplement.

**Figure 3.**
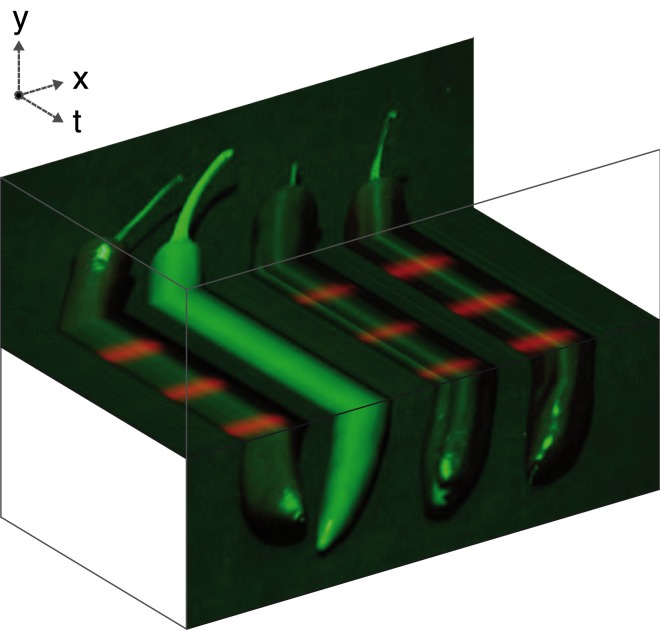
A slice through view of a neon fruit illusion video. Certain regions of the image feature a pronounced colour change from dark grey to red through time, while the rest of the image remains constant.

If you would like to prepare this illusion yourself, we present the following instructions in recipe format.

**Ingredients:**

- One or more controllable multicolour LED bulbs (e.g., LiFX A60 LED Light).
- One black table cloth.
- Fresh (or fake) fruits and vegetables of a wide range of colours.

**Recipe:**

- Take the produce and arrange to your liking on the black cloth.
- Immerse the fruits and vegetables in light emitted from the bulb’s green channel.
- Add a dash of the red channel to taste until the desired mixture is obtained and some of the fruits and vegetables are nicely reddened. A little goes a long way, and too much will overpower the dish.
- Remove the red channel component, subsequently adding and removing in a gradual manner.
- Serve immediately and enjoy. Alternatively, the dish may be video recorded with a standard camera and enjoyed another time.

For best results, use LEDs of narrowband spectra and ensure the recipe is prepared in a very dark environment. It is worth noting that the illusion is easy to set up and allows many people to experience it at the same time. For maximum surprise, consider using a set of spray-painted plastic bananas, and revealing their true colours with a white illuminant at the end. *Buon appetito!*

In case fresh fruit and vegetables, or controllable multiprimary LEDs, are unavailable, the neon fruit illusion may also be simulated. This would not be possible with standard three-channel photographs, but a good approximation can be achieved using a recently published hyperspectral database of fruits and vegetables (Ennis, Schiller, Toscani, & Gegenfurtner, 2018). The results of a lime and a red pepper, illuminated by a narrowband green LED with and without the minor contribution of a narrowband red LED, are shown in Figure 4. Although the database does not contain actual reflectance measurements, these were estimated from hyperspectral image regions taken to contain flat matte reflectance, dividing out the illuminant spectrum.

**Figure 4.**
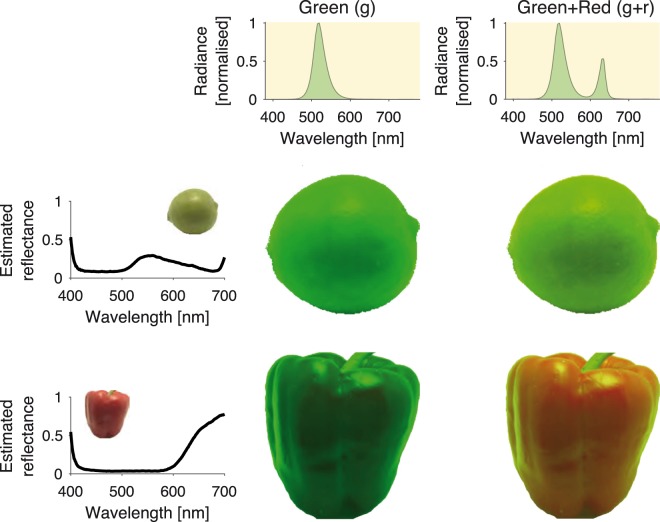
A simulation of the neon fruit illusion, using hyperspectral images of a lime and a red pepper from a database of hyperspectral images of fruits and vegetables (Ennis et al., 2018). We estimated reflectance of the lime and red pepper by dividing the spectrum of reflected light reported in the database by a measurement of the illuminant spectrum at a pixel location free from obvious specular highlights. We then rendered the lime and the repper under the illuminants given in Figure 1C using the software provided by Ennis et al. (2018).

Whether consumed fresh, preserved as a video, or simulated from hyperspectral images, the neon fruit illusion provides an engaging and surprising demonstration of colour science.

## Supplemental Material

Supplemental material for The *Neon Fruit Illusion*: A Fresh Recipe for Colour Science Demonstrations Click here for additional data file. Supplemental material for The *Neon Fruit Illusion*: A Fresh Recipe for Colour Science Demonstrations by Joshua Harvey, Takuma Morimoto and Manuel Spitschan in Perception
